# Gunshot wounds in parenchymatous organs: the morphology mainly depends on the physical properties of the affected tissues*

**DOI:** 10.1007/s00414-023-03058-2

**Published:** 2023-07-06

**Authors:** V. Thoma, G. Franchetti, D. Geisenberger, M. Glardon, J. Kromeier, K. Mierdel, S. Pollak, S. Wimmer, M. Große Perdekamp

**Affiliations:** 1 Institute of Forensic Medicine, University of Freiburg, Faculty of Medicine, Albertstraße 9, 79104 Freiburg, Germany; 2 Department of Cardiac, Thoracic, Vascular Sciences and Public Health, Unit of Legal, Medicine and Toxicology, University of Padova, Via Falloppio 50, 35100 Padova, Italy; 3 Forensic Medicine and Imaging, Institute of Legal Medicine, University of Berne, Bühlstraße 20, 3012 Berne, Switzerland; 4 Department of Radiology, St. Josef’s Hospital, Sautierstraße 1, 79104 Freiburg, Germany; 5 State Office of Criminal Investigation of Baden-Württemberg, Taubenheimstraße 85, 70372 Stuttgart, Germany; 6 Institute of Precision Medicine, Medical and Life Sciences Faculty, University of a Furtwangen, Jakob-Kienzle-Straße 17, 78054 VS-Schwenningen, Germany

**Keywords:** Gunshot injury, Parenchymatous organs, Stellate wound morphology, Liver, Spleen, Kidney, Lung

## Abstract

In contrast to gunshot wounds in skin and bone, the medico-legal literature pays little attention to the appearance of bullet penetration sites in abdominal organs. It was only in 1983 that Metter and Schulz published an article entitled “Morphological features of gunshot wounds in the liver and spleen.” According to their observations, the organs in question showed stellate tears at the bullet penetration sites resembling skin wounds from contact shots to body regions having a bony support. The study presented simulated the real conditions by means of test shots to composite models consisting of porcine organs embedded in ballistic gelatin. The ammunition used was pistol cartridges 9 mm Luger with full metal jacket round nose bullets. The shots were video-documented with a high-speed camera in order to record the bullet’s travel through the target. In addition, the composite models fired at underwent CT examinations followed by a macroscopic assessment of the organs. The study confirmed the findings of Metter and Schulz with regard to the star-like appearance of gunshot wounds in the liver and spleen. Likewise, the kidney showed radiating tears originating from the bullet path, whereas the wound track in pulmonary tissue was tube-shaped and lacked additional cracks. The varying wound patterns in parenchymatous organs can reasonably be explained as a consequence of the respective viscoelastic tissue properties.

## Introduction

Textbooks on forensic medicine and monographs on gunshot injuries usually deal with the external findings at the bullet’s entrance and exit site, the direction and range of fire as well as any conclusions about the weapon and cartridge used or the effects on the body hit (e.g. regarding a victim’s sustained capacity to act) [e.g. [1–7]]. The traumatological literature mostly focuses on the clinical wound assessment and management. In regard to the internal organs, the varying appearance and extent of tissue damage along the bullet path is rarely addressed in clinical reports [8]. According to the notion of most lay persons, the wound track is simply thought to be a tubular destruction of the perforated body parts with a constant diameter corresponding to the bullet’s calibre.

**Fig. 1:**
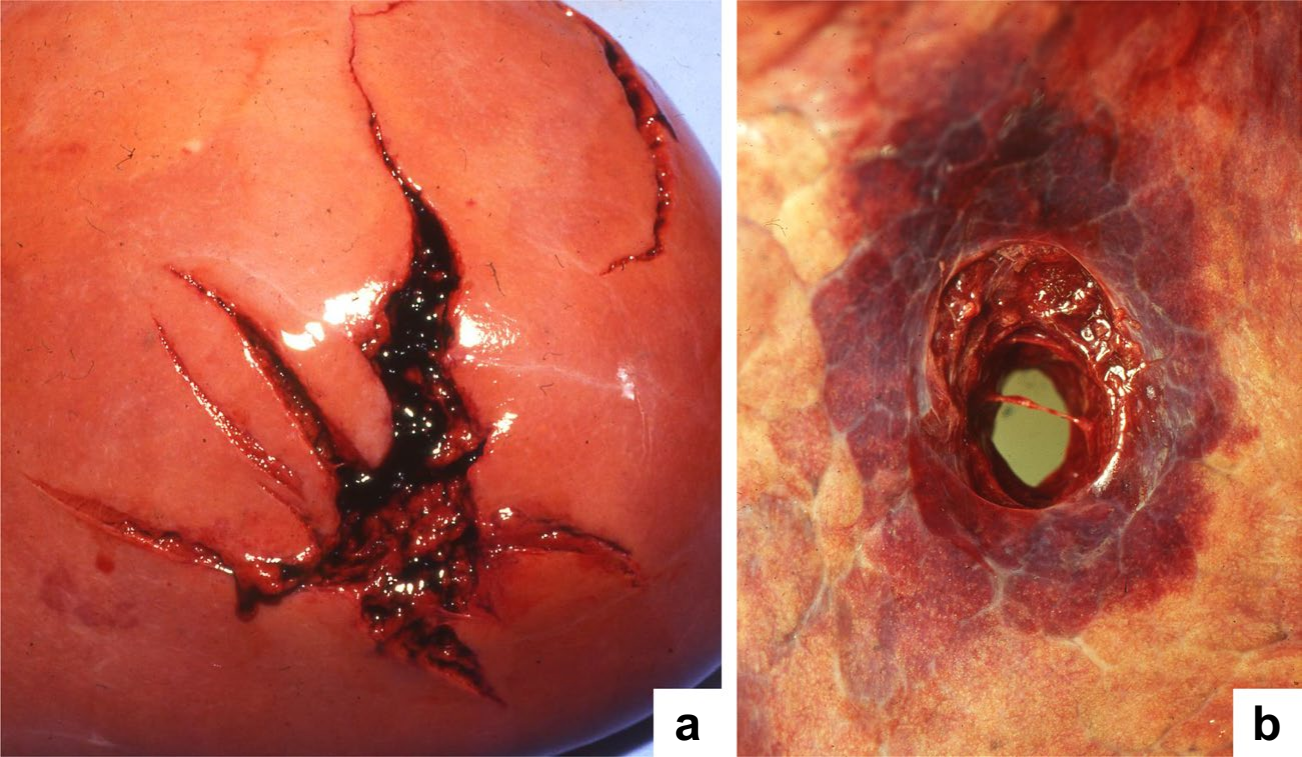
Examples of gunshot entrance sites on internal organs (autopsy findings): liver (**a**) and lung (**b**); both wounds were inflicted with pistol ammunition 9mm Luger

To the best of our knowledge, Metter and Schulz (1983) [9] were the first forensic pathologists who described the morphological peculiarities of gunshot wounds in the liver and spleen: The authors observed stellate wounds resembling the radial skin tears typically found in contact shots to body regions having a bony support. This is in line with our own observations in victims who suffered gunshots penetrating the large abdominal organs [10–12] (Fig. 1a). Apart from the liver and spleen, the kidneys show similar injury characteristics. On the other hand, the entrance and exit wounds in lungs usually lack star-shaped tears radiating from the penetration site (Fig. 1b). The different response to a specific ballistic trauma suggests that divergent physical properties of the tissues are the actual cause of one or the other wound appearance [13]. To verify the validity of this hypothesis, experimental shots were fired to porcine organs embedded in ballistic gelatin.

## Materials and methods

Composite models made from ballistic gelatin containing embedded porcine organs (liver, spleen, kidney, lung) served as targets for test shots fired from a pistol cal. 9 mm Luger. The gelatin blocks including the exenterated organs measured 20 × 20 × 30 cm. The fabrication followed Fackler’s specifications for 10% gelatin [14] and passed through the different stages as described by Kneubuehl [15].

All organs were removed from slaughtered German Landrace pigs (age 6 months, weight 110 kg). Just as in previous studies [16–19], slaughter took place for regular meat production and thus was independent of the present research project. The animal organs were used either in toto (spleen, kidney) or as a representative portion compatible with the block size (liver, lung). In any case, the organs or the resected parts were arranged in such a manner that all the tissue was covered with gelatin. Care was taken in this context that the surface areas exposed to the entering and exiting bullet were coated with the organ’s capsule (liver, spleen, kidney) or the visceral pleura (lung).

The porcine organs did not undergo any pre-treatment before being embedded in gelatin. Accordingly, the tissues were highly anaemic due to the preceding slaughtering process. The excised lung was not inflated with air so that the lobes were in a collapsed state when being shot at. The time interval between evisceration of the organs and preparation of the composite models was less than 6 h. In order to avoid heat-related protein denaturation with consecutive tissue induration and shrinkage, the gelatin had to cool down to a level just above the congealing point, which is usually below the human body temperature [20]. Only then it was poured into the casting mould with the inlying organ. Once the organs were encased in gelatin, the blocks were stored for 24 h at a temperature of 4°C.

The test shots to the composite models were fired from a semi-automatic pistol Walther PPQ M2 4” 9mm Luger (Ulm, Germany). The cartridges (MAXXTech 9mm Luger, 7.5 g, Pobjeda, Bosnia-Herzegovina) were fitted with full metal jacket round nose bullets. The shooting distance was 1.6 m and the direction of the shots was orthogonal to the front plane of the target blocks. According to the manufacturer’s specifications, the muzzle velocity of the bullets is in the order of 342 m/s.

All test shots were video-documented using a high-speed motion camera (Photron FastCam APX-RS, San Diego, CA, USA; 2,000 fps) with a view perpendicular to the direction of fire. Then the bullet entrance and exit sites in gelatin were photographed together with a metric scale so that the crack lengths could be determined without manually handling the specimens.

After being shot at, the composite models were examined on a 64-MDCT scanner (SOMATOM Definition AS; Siemens Medical solutions, Forchheim, Germany). Examination parameters: 100 kV, 750 mAs, 0.6 mm primary collimation, pitch of 0.5, system software Syngo CT VB20A. To maximize image quality all dose-saving parameters were shut off. Data was reconstructed using high contrast kernels (HR35) for the soft tissue specimens (liver, spleen, kidney) and a high-resolution kernel (HR68) for the lung specimen. Standard secondary multiplanar reformation was done in 0.6 and 3 mm slide width in soft tissue (centre/width: 35/80) and lung tissue (c/w: -400/1400) window, respectively. The VRT (Volume Rendering Technique) reformations were reconstructed using a specialized 3D suite (syngo.via, Siemens Medical Solutions, Forchheim, Germany; software version VB40B).

Subsequent to the CT examination, the gelatin blocks including the embedded organs were cut into 1 cm-slices along the whole bullet track and transverse to its longitudinal axis. The consecutive cut surfaces were photographically recorded and permitted a layer-wise determination of the crack lengths within the organs.

## Results

**Fig. 2:**
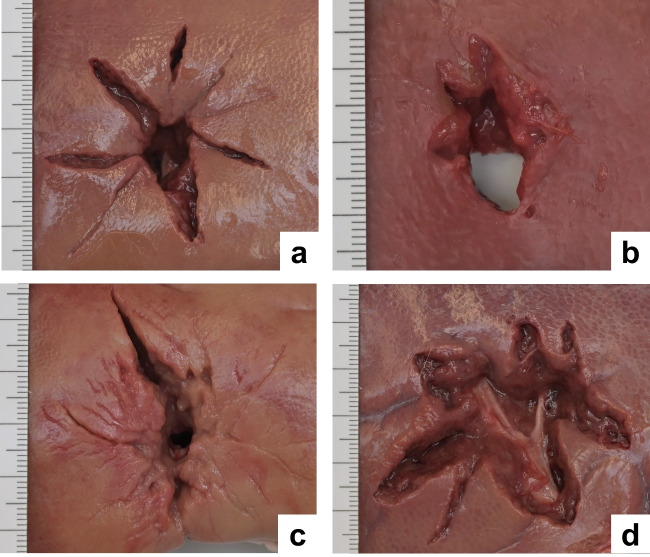
Porcine organs retrieved from the encasing gelatin after test shots. Stellate entrance sites in liver (**a**), spleen (**b**), and kidney (**c**). The findings on the exit sites resembled the bullet entrance wounds as shown by the example of the liver (d)

**Fig. 3:**
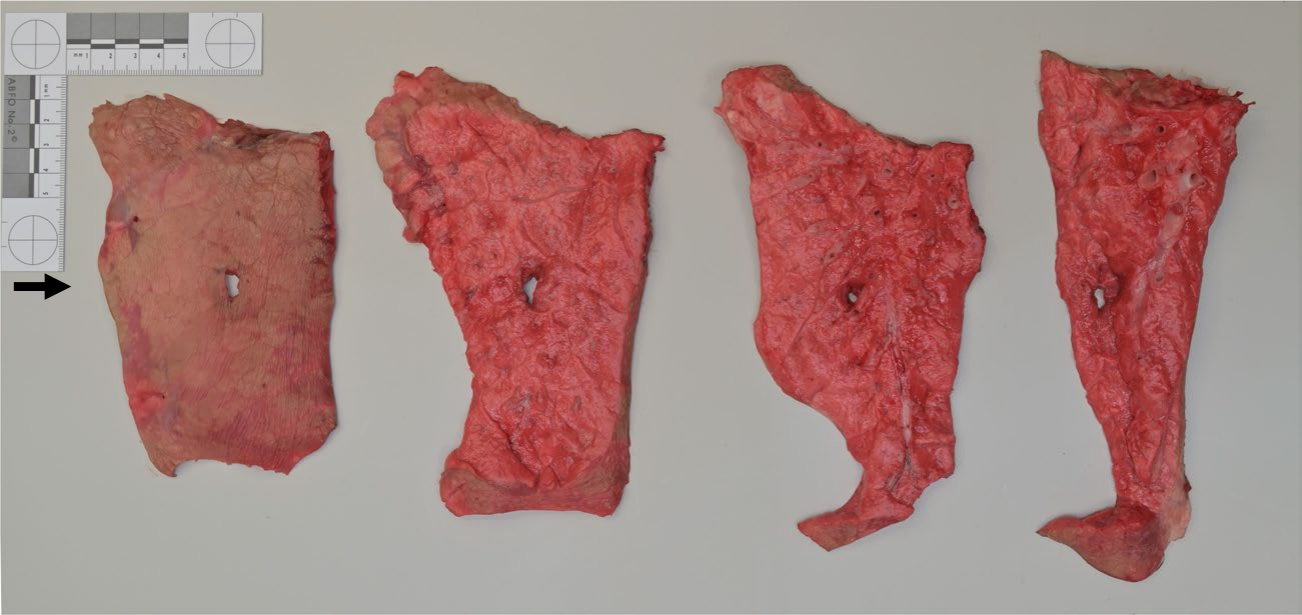
Four consecutive slices of a porcine lung specimen showing roundish tissue defects along the bullet channel. The arrow indicates the direction of shot

**Fig. 4:**
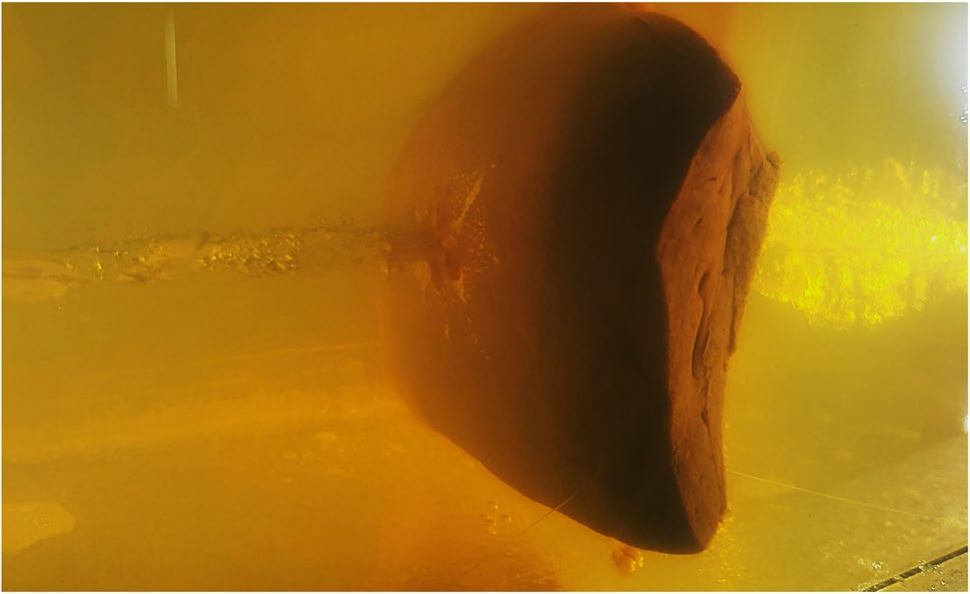
Oblique view of the liver specimen still embedded in gelatin. Note the tears radiating from the entrance hole

As expected, all test shots penetrated the gelatin blocks and the enclosed organs were perforated in full length. Therefore, the bullet entrance and exit wounds could be compared in terms of shape and dimensions. Figure 2 displays the perforation sites on the surface of the organs shot at. A stellate appearance due to radiating tears was apparent in the liver, spleen and kidney, but not in the lung sample where the tissue damage was confined to roundish holes only slightly larger than the bullet’s calibre (Fig. 3). As far as the abdominal organs were concerned, the overall size of the entry and exit wounds was in a similar order and thus did not show widening in the direction of the shot (Fig. 2d). It is remarkable that the stellate tears originating from the perforation sites in liver, spleen and kidney could clearly be seen through the transparent gelatin and thus prior to the dissection of the blocks (Fig. 4).

**Fig. 5:**
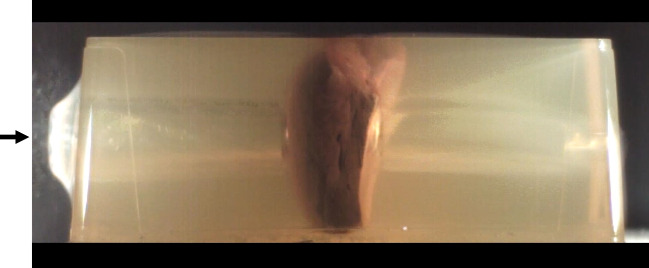
High-speed motion camera documentation displaying the temporary cavity along the bullet’s way through a composite model containing porcine liver

The high-speed videos depicted the formation and extension of the temporary cavity (TC). It had a roughly cylindrical shape characteristic for handgun ammunition with round nosed bullets [1, 21–23]. Its maximum diameter clearly exceeded the calibre of the bullet, but did not reach the dimensions known from deforming or HV projectiles (Fig. 5). Within the non-translucent organs, the development of the TC could not be assessed by direct viewing, but based on the lateral expansion of the penetrated tissue.

**Fig. 6:**
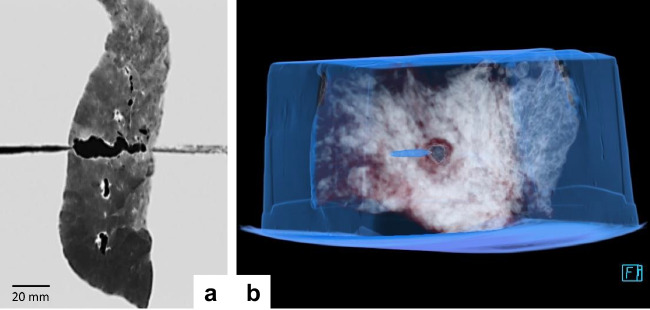
**a** Sagittal reformation of a lung specimen in 3 mm slide width using a high-resolution kernel and lung tissue window. Narrow projectile track from the entry point on the left of the screen to the lung specimen in the centre. Air-filled cavity along the intraparenchymal bullet path. **b** 3D VRT reformation of a lung specimen, looking in the direction of shot. Note the roundish entrance hole without any concomitant tissue laceration

Following the test shots, the composite models underwent CT examinations in order to record the wound morphology prior to the layerwise dissection of the blocks. Two examples (lung specimens) are given in Fig. 6.

When measuring the crack lengths on the organ surfaces, it was noticeable that the tears were not always congruent in the capsule and the underlying parenchyma. In some instances, the comparatively tight capsules were detached from the tissue below. The cut surfaces of the organ slices revealed star-like tears radiating from the bullet path and resembling the crack pattern as known from test shots to gelatin. In contrast, the wound channel through lung tissue was not accompanied by analogous tissue lacerations (Fig. 6b). Quantitative analyses of the crack lengths measured in the consecutive layers of the perforated abdominal organs were not a subject of this study.

## Discussion

Autopsy experience shows that a bullet’s wound track may change its morphological appearance on its trajectory through the body. Generally, it is to be expected that tissue destruction is most pronounced in the section where the temporary cavity (TC) had its largest extent. Besides, organ-specific textures obviously have a major influence on the interaction with the bullet and the resultant injury pattern [13]. So, it is well-known that distant shots usually cause roundish entry holes on skin. In contrast, gunshot wounds in some parenchymatous organs such as the liver, spleen, and kidney are characterized by a stellate configuration. Diverse wound manifestations might arouse doubt that they were caused by one and the same projectile. Besides, a lacerated organ injury could easily be mistaken for being the consequence of a non-ballistic blunt trauma.

It is remarkable that it took until the 1980s of the last century for Metter and Schulz [9] to publish their findings under the title “Morphological features of gunshot wounds in the liver and spleen.” The authors reported on 7 gunshot fatalities with star-like bullet entrance and exit sites in the liver and/or spleen. To the best of our knowledge, since then no systematic study has dealt with this special type of gunshot injuries. The experimental trial presented here was carried out for the sole purpose of assessing the wound phenomenology in qualitative terms without any claim to quantification. Moreover, video-documentation of the test shots should provide information on the development of the TC and the temporal sequence of tissue displacement along the bullet track.

In order to reflect real conditions, the test shots were fired to composite models consisting of organs from slaughtered pigs (liver, spleen, kidney, lung) embedded in ballistic gelatin. The latter should simulate the soft tissue surrounding the actual target organs. 10% gelatin is a well-established indicator of a projectile’s energy transfer to biological targets [15, 24–26]. Anatomy and physical properties of porcine organs are roughly comparable with those of humans [27–29].

Due to the transparency of gelatin, the dynamic interaction between bullet and simulant can be recorded by high-speed videos [23]. The cracks radiating from the trajectory mirror the original extent of the temporary cavity whose volume correlates with the energy transferred. The stellate disruptions arise from lateral displacement and circular overstretching of the simulant when penetrated by the bullet. To quantify the energy released at a particular point of the bullet path, the respective layer of the gelatin block serving as simulant can be evaluated using different approaches (e.g. by determining the sum of the crack lengths, by describing Fackler’s wound profile or by applying the polygon method [15, 24–26]). In the light of the findings obtained in the current study, the damage in ballistic gelatin—a star-shaped pattern of tears—is quite similar to the appearance of real gunshot wounds in liver, spleen and kidney.

Ruptures radiating from the bullet path indicate that the target material was temporarily exposed to tensile stress beyond the limit of plasticity. This applies to proportionally dense organs such as the liver, spleen, and kidney, but far less to the air-containing and highly elastic lung. The latter largely tolerates the momentary deformation induced by the local transfer of kinetic energy, at least in shots with non-deforming projectiles fired from conventional handguns. Accordingly, gunshot injuries of the lungs usually lack any concomitant ruptures originating from the actual bullet path. Instead, the wound morphology presents as a tubular destruction of the pre-existing pulmonary tissue. In cases of intravital infliction, the bullet channel is encircled by a concentric zone of blood extravasation (cf. Fig. 1b).

In test shots to simulants, videography reveals that the bullets rush on ahead of the temporary cavity [15, 21]. Therefore, the tissue deformation mainly takes place after the bullet has passed. As it turned out in the present study, the development and the maximum diameter of the temporary cavity were quite similar in both components of the composite models, i.e., the encasing gelatin matter, which was hit first, and the embedded organs. The displacement induced by the shot was reflected by the crack pattern in consecutive layers of the blocks. Due to the transparent nature of gelatin, the stellate configuration of the entrance and exit wounds in liver, spleen, and kidney could be seen even before dissecting the blocks (cf. Fig. 4).

Composite models made from a gelatin matrix and enclosed organs or (synthetic) bones have repeatedly been used to simulate the interaction between a bullet and a compound biological target [20, 23, 27]. By this means, it was possible to answer the question whether bone fragments act as “secondary projectiles” having the potential to create own wound channels [18]. Another point of debate was the displacement of tissue particles along the bullet path [30]. When preparing composite models, it has to be considered that gelatin must not be too hot when used for embedding soft tissues in order to prevent heat coagulation and shrinking effects [20].

All living tissues display viscoelastic behaviour, which is i. a. characterized by time-dependency of the material responses following the application of external loading. Unlike purely elastic materials, in organs and simulants these processes are delayed and impeded by internal viscous stresses [31]. Therefore, biological tissues range between elastic solids and viscous fluids [32]. Testing of the mechanical properties is complex and not within the scope of this article. Among the great number of examination techniques, the following deserve special recognition: tensile, shear and compression tests, transient elastography, ultrasound and magnetic resonance elastography [28, 33–37]. In forensic biomechanics, accurate physical characterization of organs will help to develop a complete finite element (FE) model of the human body [38]. Considering the anatomical structure, it must be borne in mind that the blood vessels supplying dense organs have a greater stretching capacity than the surrounding tissue. This is the reason why the radiate tears of stellate wounds are often bridged by intact arteries and veins (cf. Fig. 2d).

As already mentioned above, the main objective of this article is to provide a satisfactory explanation for the striking appearance of gunshot wounds in livers, spleens, and kidneys. Both theoretical considerations and test shots to composite models suggest that the radial tears along the bullet path are caused by lateral displacement and circular overstretching of the perforated tissue. Compared to the abdominal organs investigated in this study, the density of lungs is low due to their anatomical structure mainly consisting of alveoli and air ducts [31]. Accordingly, less energy is released in pulmonary tissues. The extracellular matrix of the lungs with its high content of elastic fibres provides tensile strength and elastic recoil. Thus, it is hardly surprising that gunshot injuries in lungs resemble more the bullet entrance wounds in skin than those in denser organs such as the liver.

Admittedly, the experimental parameters of the current study cannot deliver an accurate image of the conditions in reality. Although porcine organs as used in our composite models have anatomical similarities to their human analogues, the biomechanical properties can be divergent: So, it has been pointed out that porcine livers are stiffer than human ones, probably due to the different content of collagen [29]. Aside from structural varieties, the organs removed from slaughtered animals are largely drained of blood. Actually, the capsules and the underlying parenchyma would need to be considered separately. This is reflected by the fact that the crack lengths may be different in the fibrous capsules and the tissue underneath. As far as lungs are concerned, some investigators inflate the excised organs with air to a positive pressure of 20 cm H_2_O [32] before performing biomechanical tests. Regardless of the inevitable limitations, it seems remarkable that the morphological features of experimental gunshot injuries to porcine liver, spleen, kidney and lung embedded in gelatin show a good correlation with the findings in autopsy cases. It goes without saying that the study results presented here are only valid for the ammunition used, i.e. 9 mm Luger fitted with full metal jacket round nose bullets.

## Conclusions

- In shots from handguns, the entrance and exit wounds in the liver, spleen, and kidney are typically star-shaped, which is in contrast with the roundish holes seen in lung tissue.
- In the parenchymatous abdominal organs, stellate tears radiate from the actual bullet path resembling the crack pattern in ballistic gelatin.
- The varying wound morphology can be explained by the viscoelastic behaviour of the affected tissues.

## Data Availability

Data available within the article or its supplementary materials.

## References

[1] Sellier K (1982). Schußwaffen und Schußwirkungen I. Ballistik, Medizin.

[2] Lew E, Dolinak D, Matshes E, Dolinak D, Matshes EW, Lew EO (2005). Firearm injuries. Forensic pathology: principles and practice.

[3] Dodd MJ (2006). Terminal ballistics: a text and atlas of gunshot wounds.

[4] Di Maio VJM (2015). Gunshot wounds: practical aspects of firearms, ballistics, and forensic techniques.

[5] Spitz WU, Diaz FJ (2020). Spitz and Fisher’s medicolegal investigation of death.

[6] Karger B, Madea B (2022). Forensic ballistics. Injuries from gunshots, explosives and arrows. Handbook of forensic medicine.

[7] Kneubuehl BP (2022). Wound ballistics. Basics and applications.

[8] Zelder O, Koch H (1972). Gunshot injuries of large body cavities and the skull. Monatsschr Unfallheilkd Versicher Versorg Verkehrsmed.

[9] Metter D, Schulz E (1983). Morphological characteristics of gunshot wounds of liver and spleen. Z Rechtsmed.

[10] Pollak S, Saukko P (2003). Atlas of forensic medicine (CD-ROM).

[11] Pollak S, Saukko P, Siegel JA, Saukko PJ (2013). Gunshot wounds. Encyclopedia of forensic sciences.

[12] Pollak S, Madea B (2015). Schussverletzungen. Rechtsmedizin. Befunderhebung, Rekonstruktion, Begutachtung. 3.

[13] Rothschild MA, Kneubuehl BP (2022). Conventional forensic medicine. Wound ballistics. Basics and applications.

[14] Fackler ML, Malinowski JA (1988). Ordnance gelatin for ballistic studies. Detrimental effect of excess heat in gelatin preparation. Am J Forensic Med Pathol.

[15] Kneubuehl BP, Kneubuehl BP (2022). Simulants. Wound ballistics. Basics and applications.

[16] Pircher R, Preiß D, Pollak S, Thierauf-Emberger A, Große Perdekamp M, Geisenberger D (2017). The influence of the bullet shape on the width of abrasion collars and the size of gunshot entrance holes. Int J Legal Med.

[17] Giorgetti A, Große Perdekamp M, Mierdel K, Thoma V, Pollak S, Geisenberger D (2020). Arrow entrance wounds with blackened margins simulating bullet wipe. Int J Legal Med.

[18] Geisenberger D, Giorgetti A, Glardon M, Große Perdekamp M, Pollak S, Pircher R (2020). The punched-out tissue complex (skin-bone “imprimatum”) in shots from captive-bolt guns: does it act as a secondary projectile?. Int J Legal Med.

[19] Geisenberger D, Große Perdekamp M, Pollak S, Thierauf-Emberger A, Thoma V (2022). Differing sizes of bullet entrance holes in skin of the anterior and posterior trunk. Int J Legal Med.

[20] Missliwetz J, Wieser I (1986). End ballistic relation models – their use in wound ballistic research. Beitr Gerichtl Med.

[21] Vennemann B, Dautel F, Braunwarth R, Strassburger E, Hunzinger M, Pollak S, Große Perdekamp M (2008). Textile fibres along the bullet path – experimental study on a skin-gelatine composite model. Int J Legal Med.

[22] Große Perdekamp M, Pollak S, Thierauf A, Straßburger E, Hunzinger M, Vennemann B (2009). Experimental simulation of reentry shots using a skin-gelatine composite model. Int J Legal Med.

[23] Carr DJ, Stevenson T, Mahoney PF (2018). The use of gelatin in wound ballistics research. Int J Legal Med.

[24] Schyma CW (2010). Colour contrast in ballistic gelatin. Forensic Sci Int.

[25] Schyma C, Madea B (2012). Evaluation of the temporary cavity in ordnance gelatin. Forensic Sci Int.

[26] Schyma CWA (2020). Ballistic gelatin – what we see and what we get. Int J Legal Med.

[27] Große Perdekamp M, Pollak S, Thierauf A (2011). Composite models simulating soft tissue targets in experimental ballistics. Fol Soc Med Leg Slov.

[28] Yang C, Yin M, Glaser KJ, Zhu X, Xu K (2017). Static and dynamic liver stiffness: An ex vivo porcine liver study using MR elastography. Magn Reson Imaging.

[29] Estermann SJ, Förster-Streffleur S, Hirtler L, Streicher J, Pahr DH (2021). Comparison of Thiel preserved fresh human, and animal liver tissue in terms of mechanical properties. Ann Anat.

[30] Große Perdekamp M, Vennemann B, Mattern D, Serr A, Pollak S (2005). Tissue defect at the gunshot entrance wound: what happens to the skin?. Int J Legal Med.

[31] Suki B, Stamenović D, Hubmayr R (2011). Lung parenchymal mechanics. Compr Physiol.

[32] Dai Z, Peng Y, Mansy HA, Sandler RH, Royston TJ (2015). A model of lung parenchyma stress relaxation using fractional viscoelasticity. Med Eng Phys.

[33] Arda K, Ciledag N, Aribas BK, Aktas E, Köse K (2013). Quantitative assessment of the elasticity values of liver with shear wave ultrasonographic elastography. Indian J Med Res.

[34] Mulabecirovic A, Mjelle AB, Gilja OH, Vesterhus M, Havre RF (2018). Liver elasticity in healthy individuals by two novel shear-wave elastography systems – Comparison by age, gender, BMI and number of measurements. PLoS One.

[35] Li J, Venkatesh SK, Yin M (2020). Advances in magnetic resonance elastography of liver. Magn Reson Imaging Clin N Am.

[36] Trout AT, Anupindi SA, Gee MS, Khanna G, Xanthakos SA (2020). Normal liver stiffness measured with MR elastography in children. Radiology.

[37] Yin M (2020). Normal liver stiffness values in children are the same as in adults. Radiology.

[38] Umale S, Deck C, Bourdet N, Dhumane P, Soler L (2013). Experimental mechanical characterization of abdominal organs: liver, kidney & spleen. J Mech Behav Biomed Mater.

